# Men’s health, physical activity, and ageing in a professional football club community organization programme

**DOI:** 10.1093/heapro/daaf204

**Published:** 2025-12-02

**Authors:** Tom Duffell, David Haycock, Andy Smith

**Affiliations:** Department of Secondary and Further Education, Faculty of Education, Edge Hill University, Ormskirk L39 4QP, United Kingdom; Centre for Mental Health, Sport and Physical Activity Research, Edge Hill University, Ormskirk L39 4QP, United Kingdom; Centre for Mental Health, Sport and Physical Activity Research, Edge Hill University, Ormskirk L39 4QP, United Kingdom; Faculty of Education, Edge Hill University, Ormskirk L39 4QP, United Kingdom; Department of Secondary and Further Education, Faculty of Education, Edge Hill University, Ormskirk L39 4QP, United Kingdom; Centre for Mental Health, Sport and Physical Activity Research, Edge Hill University, Ormskirk L39 4QP, United Kingdom

**Keywords:** age, communities, masculinity, professional sport, social determinants, walking football

## Abstract

Professional sport club community organizations (CCOs) are regarded as important neighbourhood settings for men’s health promotion. However, little is known about how age, and perceptions of ageing and masculinity, shape physically inactive men’s engagement in CCO-led health programmes. This study addresses this international knowledge gap by presenting novel insights on the lived experiences of 17 physically inactive men (aged 30–71) involved in the Active Blues programme based in north-west England. Men who engaged in the programme initially regarded it as an important opportunity to return to competitive sport. However, it was the facilitation of traditional masculine values of banter and camaraderie with new like-minded peers which was essential for men’s continued engagement, and which helped them manage age-related health conditions. Those who did not engage in Active Blues were more likely to have accepted socially constructed deficit-focused narratives of ageing which, for some, meant ‘slowing down’. Fulfilling family commitments and caring responsibilities also prevented some men’s engagement. The findings emphasize the need to develop more diverse and culturally nuanced understandings of engaging men in gender-responsive CCO-led health promotion programmes which are underpinned by conceptions of positive, strengths-based, healthy masculinities and which reinforce notions of peer support, camaraderie and openness.

Contribution to Health Promotion

- The research reports novel insights on the diversity of men’s ageing experiences and perceptions of engaging in CCO-led health programmes.
- Men who engaged in the programme initially regarded it as an opportunity to return to competitive sport, but its social nature and facilitation of traditional masculine values (e.g. banter) was more important for long-term engagement.
- Men who did not engage were more likely to accept socially constructed deficit-focused narratives of ageing which meant ‘slowing down’ rather than being active.
- CCO-led health promotion programmes might help promote physical activity, but cannot overcome the deep-seated inequalities underpinning the social determinants of health.

## INTRODUCTION

The role of professional sports clubs and their charitable or community organizations (sometimes known as club community organizations, CCOs) in tackling health inequalities has been increasingly emphasized as one key aspect of the convergence of public health and community sport policy (Mansfield 2016, Smith *et al*. 2022, Duffell *et al*. 2023). Although typically independent of their associated professional club (e.g. in terms of their governance, funding, staffing and strategic priorities; see Sanders *et al*. 2021), in many countries, including the United Kingdom, CCOs are now viewed as important vehicles of intersectoral public health promotion in neighbourhoods and communities, and as especially ideal settings for the prevention of noncommunicable diseases (NCDs) and promotion of mental health (Wyke *et al*. 2015, Curran *et al*. 2017, Brazier *et al*. 2025a, 2025b, Maclean *et al*. 2025). It has been argued that the brand of professional sport (including football) can be an important ‘hook’ for engaging and appealing to broad groups of local residents, especially men and those living in under-served communities (Pringle *et al*. 2013, 2021 , Hunt *et al*. 2014 , Wilcock *et al*. 2021, Brazier *et al*. 2024, Maclean *et al*. 2025). Evidence has shown that the many programmes delivered by CCOs—which vary considerably in terms of their funding, organizations and personnel involved, and approaches to monitoring and evaluation—have contributed to various positive health outcomes among men. These include long-term weight loss (e.g. Wyke *et al*. 2015, Gray *et al*. 2018), improved mental health and suicide prevention (e.g. Benkwitz and Healy 2019, Dixon *et al*. 2019, Wilcock *et al*. 2021), increased quality of life among those living with cancer (e.g. Rutherford *et al*. 2021), and reduced risk of various cardiovascular disease factors (e.g. Pringle *et al*. 2014, Robertson *et al*. 2016, Zwolinsky *et al*. 2016).

Notwithstanding the rapid growth and increasing sociocultural appeal of programmes delivered by professional sport CCOs in engaging so-called ‘heard-to-reach’ men, other studies have shown that there exist many challenges in the effective, scientifically robust, monitoring and evaluation of such programmes (Brazier *et al*. 2024, 2025a). These challenges include: the degree to which organizations are committed to evaluating their work, and if so, whether they have the requisite skills and expertise needed to do so; the often short-term nature of relationships with evaluation partners; a lack of a standardized impact and outcomes frameworks to inform monitoring and evaluation approaches; knowledge of the health behaviours and outcomes to be examined; and availability of funds and resources to support evaluation (Brazier *et al*. 2024, 2025a). It has also been reported that while CCOs can be viewed by stakeholders as important assets in local health promotion, knowledge of their work often varies, programme rationales can be narrowly understood by health partners as relating primarily to increased sport participation rather than health promotion, and the motivations of CCOs are sometimes questioned given perceptions of their close association with professional sports clubs and their various corporate social responsibilities (Brazier *et al*. 2025a). Whether CCOs are connected with, and embedded in, local health ecosystems and have sufficient capacity to collaborate with other organizations with shared priorities has also been shown to compound the many other challenges involved in maximizing the impact of CCO programmes on the health of local residents (Pringle *et al*. 2021, Brazier *et al*. 2025a, 2025b).

The challenges of evidencing the impact of CCO-led public health (e.g. food hub service, physical activity) and education (e.g. curriculum vitae writing, employment preparation, citizen’s advice) programmes, and securing funding to sustain these, was identified in another qualitative study involving 24 staff members from the CCOs of 22 professional football clubs and one nonprofessional club in Scotland (Maclean *et al*. 2025). The participants recalled having safeguarding and staff/volunteer training concerns when supporting people experiencing significant social and health challenges, and reported how good partnership working between the CCO and professional club was important, but concerns were often raised about the blurring of these organizational boundaries. This was especially the case in relation to whether ‘the branding of the club could overshadow the work of the community arms, or that they would be perceived as one and the same’ (Maclean *et al*. 2025: 5). Variability in the size and kind of staffing available between CCOs, and the facilities they could access to deliver programmes, also constrained the scale and impact of social and public health programmes delivered by CCOs in Scotland (Maclean *et al*. 2025).

Important though these previous studies are, if we are to better understand whether CCO-led health programmes can engage and improve the health of ageing men, it is important to examine their experiences of these in more detail. However, as Thomas and Thurnell-Read (2024: 190) have noted, the ‘experiences, identities, and practices of ageing men are under-researched’. The present study therefore extends the previous work cited above by exploring how age, and perceptions of ageing and masculinity, helped shape whether physically inactive men aged 35–50 living in under-served communities engaged, or did not engage, in the CCO-led health programme, ‘Active Blues (AB)’. Such an approach not only helps to advance international understanding of how, and in what circumstances, physically inactive men do or do not engage in neighbourhood-based health promotion programmes delivered by CCOs. It also raises a question of fundamental importance in public policy terms: to what extent can CCO-led programmes be used to increase physical activity and improve the health of men given their perceptions of ageing, masculinity, and the social circumstances in which they live?

## MEN’S HEALTH, MASCULINITY AND AGEING

It is now well-established from international evidence that men (especially those in under-served communities) and men’s health are in need of urgent attention. For example, in announcing its call for evidence to inform a new Men’s Health Strategy for England, the UK Government’s Department for Health and Social Care summarized the latest evidence which indicated that men in England live on average four fewer years than women, and three in four people who die by suicide are men (DHSC 2025, see also, Galdas *et al*. 2025). Men also continue to be disproportionately more likely to die from various NCDs (e.g. cardiovascular disease, Type 2 diabetes, cancer), and engage in unhealthy lifestyle behaviours (e.g. smoking, harmful gambling, excessive alcohol consumption, substance use), with those living in the most under-served communities being more likely to die earlier (DHSC 2025, Galdas *et al*. 2025). Although CCOs have proven effective in engaging men at risk of future ill-health in health promotion programmes (Pringle *et al*. 2013, 2014, Wyke *et al*. 2015, Gray et al. 2018), many men continue to be more reluctant to access traditional health services and to seek help when they experience ill-health than women (Galdas *et al*. 2005, Oliffe *et al*. 2020, Galdas *et al*. 2023, 2025, Sharp *et al*. 2024). That being said, as Galdas *et al*. (2005) have noted, not only are occupational and socio-economic status more important variables (among others, such as age) than gender alone, men are not a homogenous group and more attention needs to be paid to how various forms of masculinities and men’s perceptions shape their willingness to seek help when experiencing symptoms of ill health (see also, Galdas *et al*. 2023, 2025). More recently, a scoping review by Oliffe *et al*. (2020) concluded that men’s health literacy is not only equally important for understanding men’s experience of seeking help for their health, but also their engagement with community-based men’s health promotion programmes (including those provided by CCOs). No less importantly, they add, is the need to better understand ‘how socially constructed masculine identities, roles and relations connect with men’s health literacy and inequities’ (Oliffe *et al*. 2020: 1048), and other identity markers including age.

In England, the significance of age for men’s health is brought into even sharper relief given the impacts of a continued ageing population. It is currently estimated that, between mid-2022 and mid-2032, the number of people at state pension age (currently 65, but planned to rise to 67 for both sexes) will increase by 1.7 million to 13.7 million people (a 13.8% increase), and by mid-2047 the proportion of people aged 85 years and over is expected to have nearly doubled to 3.3 million, or 4.3% of the total UK population (Office for National Statistics 2025). One of the parallel concerns about an ageing population are declining physical activity levels and associated rises in illness, frailty, disability, loneliness and social isolation (Dionigi 2006, 2015, Mansfield *et al*. 2019). Indeed, this traditional ‘deficit’ model of ageing which is commonly articulated in policy and other aspects of public life suggests that ageing almost inevitably results in an unavoidable decline of health (Blaikie 1999, Pike 2011a, 2015, Gard *et al*. 2018), and is a process in which we experience deficits, disease and other age-related problems requiring intervention and/or treatment (Pike 2019). This narrative reinforces the perception that poor health can be managed by accepting personal responsibility and making positive healthy lifestyle choices, including the promotion of physical activity as part of the active ageing agenda (Lupton 1999, Pike 2011a, 2011b).

The active ageing agenda promotes a return to physical activity (including through CCO-led programmes) as being important for maintaining youthfulness and reducing the burden on social and health care services (Bytheway 1995, Vincent 2003, Gard *et al*. 2018). Although such an approach arguably unnecessarily problematizes older people (Pike 2011a), engaging in community-based programmes such as those delivered by CCOs can help challenge stereotypical and stigmatizing perceptions of older people (including men) as being a burden and dependent (Pike 2011b, 2015). Indeed, in his study of walking football participants in the United Kingdom, Thomas (2024) suggested engaging in walking football enabled older people to form friendships and a sense of community, care and collectivity, and create identities which challenged assumptions of loneliness and degradation. Walking football also allowed the participants to see themselves as being physically and mentally active, and as having a sense of purpose and positive future outlook—albeit in ways shaped by their intersectional experiences and other structural constraints on their participation and health (Thomas 2024, Thomas and Thurnell-Read 2024, see also, Raisborough *et al*. 2014, Dionigi and Son 2017).

The additional potential benefits of engaging adults and older people via CCO-led programmes were outlined in a 3-year evaluation of the Extra Time Hubs pilot programme funded by the English Football League Trust and facilitated by eleven CCOs (Bullough *et al*. 2023). The local hubs were designed to increase social connections which may lead to engagement in physical activity among those aged 55 and above, and which create the conditions for participants to engage in off-shoot activities outside of formal programme sessions activities. The hubs typically engaged older groups than expected (an average of 71 when joining), more women (53%), and almost two-fifths classed themselves as having a long-lasting disability (38%), as living alone (37%), and as being physically ‘inactive’ (less than 30 minutes per week) (Bullough *et al*. 2023). The findings revealed that the hubs had a greater impact on participants’ mental wellbeing compared to their physical activity levels or motivation to be active, and those who were least active at baseline were more likely to remain engaged in the programme for longer (Bullough *et al*. 2023). The hubs were also reported as being especially important for improving social connections, reducing loneliness, and providing opportunities to meet friends regularly which they looked forward to (Bullough *et al*. 2023). In this regard, the authors concluded that the community hub concept was a relatively low-cost solution which enabled CCOs to become part of the local health ecosystem to support the health of older people. This was strengthened by the CCOs’ ability to engage participants through ‘the power of the football badge’ and ‘the attraction of attending the stadium for gatherings [which] was, for many, a key part of their initial engagement with a Hub’ (Bullough *et al*. 2023: 3).

## MATERIALS AND METHODS

### About active blues

AB was a community-based CCO-led programme funded by Sport England in the second round of its ‘Get Healthy, Get Active’ initiative (Duffell *et al*. 2023), the design and delivery of which was examined in a wider multi-method, cross-sectional, study from which the data reported in this paper were derived (see Duffell 2019, Duffell *et al*. 2023). The project received institutional ethical approval from Edge Hill University.

AB was evaluated by Edge Hill University researchers and delivered by Everton in the Community (EitC) (the official charity of Everton Football Club) in four electoral wards throughout North Liverpool which were located in some of the most under-served and low-income communities in England. The primary outcome was to support inactive men aged 35–50-years-old to become physically active at least once per week through participation in sport. Secondary outcomes included enabling men to adopt healthier lifestyles and reduce health inequalities that lead to Type 2 diabetes, musculoskeletal conditions, obesity, isolation and loneliness, poor mental health and cardiovascular disease.

Participants engaged in at least one self-selected weekly sport session from various activities (including walking football, yoga, gym-based sessions) delivered over three week days by EitC staff. Sessions were delivered in various sports facilities based at CCO locations (e.g. outdoor 5-a-side pitches, indoor sports hall, gym, fitness studio).

### Recruitment and participants

The wider study involved 67 men from two populations who engaged in group interviews: (i) ‘AB’ participants (*n* = 27), which consisted of men who had attended weekly sport sessions for a minimum of 4 weeks at the time of interview; and (ii) men (*n* = 40) who chose not to attend AB sport sessions but had participated in one of five initial engagement events for the programme called Lads Night In (LNI) which were held at Goodison Park, the former home stadium of Everton Football Club. The LNI events informed men about ‘AB’ in a nonthreatening and engaging way using various Everton-themed activities. These included talks with former players, photo opportunities with replica cups, and opportunities to win signed memorabilia. Food and drink were also provided, and former players of a similar age to the target audience actively promoted the programme.

### Data generation

In this paper, we focus on 17 participants (11 AB, 6 LNI) of the wider sample of 67 men who consented to take part in follow-up individual semistructured interviews between June 2016 and December 2017. The AB participants were aged between 30 and 71, and five lived in communities ranked as being in the 10% most deprived in England (Table 1). The LNI participants were aged between 32 and 56, and all but one lived in areas ranked in the top 10% most deprived nationally (Table 2). All participants were self-defined as White, heterosexual, and nondisabled. The names of participants quoted in the paper are pseudonyms and ethical approval for this study was granted by Edge Hill University’s Research and Ethics Committee.

**Table 1.** AB participants.

Name	Age	Post code deprivation^a^
Alistair	64	40% least
Simon	36	10% most
Sam	71	10% most
James	49	40% most
Chris	58	30% least
Ben	40	10% most
Jonathon	30	10% most
Phillip	61	30% most
Thomas	54	40% most
David	43	20% most
Alfred	64	10% most

^a^Based on English indices of deprivation [Ministry of Housing, Communities and Local Government (MHCLG), 2015] .

**Table 2.** LNI participants.

Name	Age	Post code deprivation^a^
Peter	53	10% most
Arthur	56	10% most
Daniel	32	10% most
Jack	33	10% most
Alan	40	10% most
George	49	50% most

^a^Based on English indices of deprivation (MHCLG, 2015).

Semistructured interviews lasted between 30 and 60 minutes, and were held in a quiet room at either Goodison Park or the Everton Free School (a venue where AB weekly sessions were held). All interviews were audio recorded with the prior verbal and written consent of participants. Interviews enabled a more detailed exploration of emerging lines of enquiry generated by the group interviews, participants’ individual experiences and feelings (Braun and Clarke 2020, 2021) about AB and LNI, and especially why participants had decided to engage in AB or not. It also enabled us to explore interviewees’ experiences of physical activity and health, how these were shaped by intersectional characteristics (especially age and gender) and their perceptions of masculinity, and the effectiveness of the brand of professional football in helping to engage physically inactive men in a CCO-led programme.

### Data analysis

All interview recordings were transcribed verbatim before being analysed thematically in line with the approach outlined by Braun and Clarke (2020, 2021). Firstly, the first author conducted a familiarization of the data set by reading through all transcripts as part of an initial search for potential codes and patterns of behaviour identifiable in the data. Secondly, the first author undertook the coding process on hard copies of the transcriptions before applying these codes in the NVivo 11 software programme to help manage the data set. During this stage, deductive ‘*in vivo* codes’ or words and phrases used by the participant (e.g. ‘Everton’, ‘funding’, ‘brand’, ‘partners’), and inductive ‘analytic codes’ relating to the research questions posed and underpinned by key theoretical concepts (e.g. ‘interdependency networks’, ‘power’), were identified. The researcher-derived codes across all transcripts were subject to a cross-checking process by the second and third authors, before candidate themes were developed which included ‘age as a motivator for, or barrier to, activity’, ‘managing my health’, and ‘banter, blokes, and family’. These candidate themes were discussed by all authors before being revised again until the final themes were identified: (i) the paradox of age and activity; (ii) getting active as a form of health management; and (iii) ‘It’s because of the lads’ (and family): the importance of social relations (see Table 3).

**Table 3.** Thematic structure.

Theme title	Summary	Subthemes
The paradox of age and activity	Participants shared how their perception of age/ageing influenced participation in ‘AB’	Age as a motivator for, and barrier to, activity (Age) Appropriateness of ‘AB’ (‘AB’—Football, intergenerational and competition/’LNI’—Individualized, too old or too young)
Getting active as a form of health management	Participants explained the importance of their health or how poor health inhibited participation	Health as a motivator for, and barrier to, activity Managing my health
‘It’s because of the lads’ (and family): the importance of social relations	Participants described the importance of social relations and how this played a key role in their decision making	Socializing with like-minded men Banter, blokes and family

## RESULTS

### The paradox of age and activity

Many of the men recalled positive previous experiences and memories of playing sport (especially football) and described it as being ‘like going back to childhood and standing there like a ten-year-old’ (Ben, AB). Attending AB provided men with an opportunity to play sport again, even though their enthusiasm was initially tempered by concerns about ‘their age’. For example, Alfred (AB) explained that despite having doubts about age, his desire to play football helped him to overcome these doubts:

I wouldn't say I was tense, but I wasn't relaxed as the way I am now. I think I had too much energy that I needed to burn…Just to have a game of football again. You know, at 64 I thought that it was out of my reach, and now [having engaged in Active Blues] I feel like a bit of a kid in a way.

AB participants also viewed other obstacles, such as health conditions, as motivators to become more physically active and used their health goals to sustain their engagement with the programme even though some expressed concerns about ‘slowing down’ as they aged. As Phillip (AB) explained:

As you get older you slow down, you can't do the things that you used to be able to do, so it [Active Blues] just gives you an opportunity to get active again, and health wise, I've lost just over a stone.

James (AB) also found that turning age 50 was a trigger for reflecting upon his own health, particularly given that his work environment was one where he often encountered individuals with declining health:

I'm fifty this year…I'm looking round at the patients in our waiting room, seeing them come in as newly diagnosed diabetics, and it makes you start to think about yourself a bit more is the honest answer. And I think I must have been looking for something [like *AB*] to start.

The appeal of the socializing and bonding opportunities with men of a similar age and ideas was also articulated by other men who engaged in AB, including (Alistair, AB), who explained how the activities provided as part of the programme enabled him to ‘(get) out of the seat and doing the activities, some of them a bit structured, and then meeting people the same age, similar age, with the same ideas’.

In contrast to the experiences recalled by AB participants, those LNI attendees who did not engage in the programme typically perceived age differently, and often as a barrier to returning to sport. Indeed, LNI participants saw sport as something that people ‘their age’ did not do and that as they aged they should be ‘slowing down’. This was a view articulated clearly by (George, LNI) who said: ‘[I’m]a bit more achy…I'm getting older, you know… It's time to start slowing down a bit…You'll know when you get older yourself’. Age-related declines in health was also part of the reason why other LNI participants felt that they were not as capable as they once were. As Peter (LNI) put it: ‘After fifty you’re knackered’. Another, George (LNI), explained how his experience of a heart attack aged 47 resulted unintentionally in his declining physical activity which meant that he was unlikely to engage in AB. He said:

At about forty-seven…I had the heart attack…I'd started settling down…I suppose it just tailed away [being active]. It just happens, doesn't it? It's not intentional, but it happens.

Unlike the AB participants who engaged in the programme even if their age fell outside of the advertised target range (aged 35–50), the LNI men reported that marketing the programme in this way dissuaded them from engaging in the programme, especially if they were older than the upper age limit. For example, Daniel (LNI) who was aged 32, explained that:

It could appeal more to certain ages [older people], but there's got to be certain factors [like competition] in there for your age [31]. It's how you market that. There's got to be something [appropriate for my age group], because there's a big gap between the lowest age and the top age.

Other LNI men perceived EitC as a charity that only delivered programmes for younger people, rather than anything that was appropriate for men of his age to attend: ‘I think with something like that [AB], it's sort of aimed at younger people …I'm too old…Just you don't think of them [EitC] doing things with…older people’ (participant LNI).

### Getting active as a form of health management

Most AB participants felt that engaging in the programme enabled them to derive positive health and fitness benefits from being active and reducing the time they spent being sedentary, as James (AB) explained:

The first thing is getting fitter and getting out there and not just [being] sat in front of the tele all evening. I think that's the key thing, and just the feeling good about doing it as well, and that psychological side to it, which I never expected anything like that really, with exercise.

This was also recognized by Thomas (AB) who explained why he engaged with AB as follows: ‘I just think the fitness aspect first of all, and just to keep doing something’. In addition to improvements in fitness, weight loss was identified as another reason for, and benefit of, engaging in the programme by other participants:

So it's a whole thing with me. I feel better, and again, it's a smaller tee-shirt and that now, and I've still got quite a bit to lose, but I'm well on my way. I've lost a good bit of weight. Simon (AB)

Other men cited weight loss, lower blood pressure and reduced stress as important health benefits which meant they continued to engage in the programme. For example, Chris (AB), said:

It [weight] went down well, but…it meant all my clothes had to be thrown out. I had to buy a new set of clothes, but that's a different matter. So just the benefits from that, really, and then all the exercise did ease the stress, and that's gone. I had high blood pressure too.

Alistair (AB) similarly described how engaging in AB had helped mitigate the impacts of leading a sedentary lifestyle:

The benefits of getting off your backside and getting out of the seat, because I have lost…I've put a bit back on, but I lost two stone, and I lost eleven centimetres on my waist…And the heart rate came down, and cholesterol. It all improved a lot.

AB appeared to positively impact health conditions often managed through lifestyle intervention with Phillip (AB) describing how the management of his type II diabetes had improved as a result of his continued attendance, stating:

I'm a diabetic, type two, and that's gone a lot better. My insulin sugars, yes. My sugar level's gone a lot better, and it's more active. It's a lot better.

In a similar manner to those men who engaged in AB, some LNI participants also recalled being physically active because of previous health problems but did not want to regard AB as an appropriate setting for this. Jack (LNI) also said:

I’d rather do something else. I’m happy with doing my Wii, and we’ve got an exercise bike at home so I pedal on that.

Other men, including Arthur (LNI), described how their health problems prevented him from participating in AB:

I've just started going the gym, because M's [Son] got a wristband because he's disabled, so I've been going. I occasionally go with him when I'm off work, but the problem I have is, I have to ensure that I don't overdo it, because the next day I suffer for it. And one of the other problems I have, I have diabetes, which I have to take medication for, and I have problems with my feet.

### ‘It’s because of the lads’ (and family): the importance of social relations

All AB participants reported that having the opportunity to socialize with other like-minded men was another reason why they continued to engage in the programme. The men referred frequently to the ‘banter’ and ‘camaraderie’ between those who attended the sessions and did not anticipate this having such a profound effect on their engagement at the outset of the programme. David (AB) explained why he continued to attend AB thus: ‘Initially it [involvement in the programme] was for fitness. Maintaining it [involvement in the programme], it's because of the lads’. Thomas (AB) also made reference to the ‘banter’ among the other men who attended the sessions and described how this created an enjoyable environment for those who attended: ‘Really just the banter and that with all the blokes and that, and we've all got to know each other, and we have a really good laugh and that’.

The appeal of the socializing and bonding opportunities provided by AB was articulated by other men, including Thomas (AB) who discussed the importance of meeting new people and how he has enjoyed the company of others who attend weekly walking football sessions:

The company's good, you know what I mean? The company is excellent. That's a big, big part of why I keep coming, you know, because I'm meeting people and they're meeting me, and we're enjoying something we all enjoy, and it's going really, really well. I'm delighted with it, I'm made up.

The particular value placed upon meeting friends was also described by Sam (AB) as a vital component to his continued involvement. He described how football was something that he has always enjoyed, but that socializing with his ‘decent, good friends’ at AB was important to him: ‘I'm coming here doing something I've always enjoyed…it's getting me out of the house…I've met a lot of decent, good friends, in here as well’.

Football was often cited as the context in which ‘banter’ could be created and, as Ben (AB) noted, was something that was present in football dressing rooms when he last played competitively for a team. In particular, playing football (and the associated dressing room environment) was regarded as providing a male-exclusive escape from work environments where females were often present:

It's keeping me active, and I enjoy it. I enjoy football. As I say, I enjoy the banter with the lads. You miss the dressing room. When you've played in a team, you miss the dressing-room. Old footballers’ll tell you that, professionals. They miss that, and comradeship. I work with a lot of women, which is great. Don't get me wrong, that's lovely, but I don't get much male company, and male company, the banter, is funny.

Many of the men engaged in AB were supporters of football teams, but regardless of their chosen allegiance, they felt welcomed into the established networks the men had developed on the programme. Indeed, although the majority of men supported Everton, those who supported rival teams such as Liverpool, also contributed to the enjoyable ‘banter’, and the fact that it was an exclusive male preserve. As James (AB) explained: ‘I enjoy a bit of banter with the lads. Most of them are Evertonians, and if not, they're Liverpudlians. They're a nice minority, like’.

Important age-related social relations also played a key role in the decision of whether to attend AB for LNI participants. Specifically, they often discussed how family and caring responsibilities were reasons why they were unable to attend. It was important for Peter (LNI) that he prioritized looking after his family and house over attending sport-based programmes like AB:

Family first, isn't it? Family, I've got to look after my family, my house. I've got to keep everything close to me first before I start doing stuff like that, but then if I do get the chance, I would have a go.

He reiterated that it ‘was a given’ that spending time with his family would come before anything else and this meant he was unable to attend the programme:

My family will always be first. I'd always put them first. That's a given. But if it's something like it's once a week or once a fortnight [I might go]…I couldn't do it three times a week, I simply couldn't.

Family commitments also played a factor in Arthur (LNI) decision to not engage in the programme. His family commitments also included caring responsibilities which he explained would present challenges to attending, but he was more open to the opportunity if he was able to take his son and if the programme was inclusive for those with disabilities:

If I could get [my son] involved. I just want to try and include him in this community…[my son] has got cerebral palsy, which affects his left side, so he has to wear a splint…He had a slight epilepsy, which is under control, but the physical side, it's very, very tiring for him, and I thought, if that's something I could come along to, then he could come with me, and he'd enjoy it, and more importantly, it's with Everton.

Similar challenges were also encountered by George (LNI) who was a carer for his mother:

I've had to come out of work and become my Mum's carer…I'd have to say, well, if it's on a Monday and a Wednesday and a Friday, I'd have to fit it round [a carer]. There'd have to be somebody there for my Mum…even if it's only a couple of hours in the day.

## DISCUSSION

Our findings emphasize the need to recognize men’s diverse experiences and perceptions of engaging in CCO-led programmes intended to promote their engagement in physical activity and generate other positive physical and mental health outcomes. All participants contextualized their experiences—as men—of AB in relation to age and ageing, and in so doing attached multiple meanings to masculinity and health which were expressed through their engagement, or not, in AB. Those participants who attended AB typically resisted socially constructed deficit-focused narratives of ageing and associated stigmatizing perceptions of decline and dependency (Pike 2011b, 2015, Thomas 2024) by regularly engaging in walking football. They viewed AB as providing a platform in which they could age successfully, often referring to overcoming age-related health conditions, either in terms of managing or avoiding them in the future (Pike 2019, Thomas 2024, Thomas and Thurnell-Read 2024). For the men who engaged in AB, walking football provided them with an opportunity to play competitively, develop new friendships, strengthen their sense of self, and engage in opportunities for peer support and bonding with like-minded men (Sharp *et al*. 2024, Thomas 2024, Thomas and Thurnell-Read 2024). Since they played running football in their youth, for these men, AB provided an opportunity to engage competitively again in walking football as part of ‘a ‘revitalization’ of their youth’ (Thomas 2024: 12). In so doing, the programme was a setting in which men had ‘a chance to cultivate and display physical prowess’ and where their ‘embodied performances were vital for cultivating a masculine identity which, whilst threatened by the ageing process, sustained their privilege and status’ (Thomas and Thurnell-Read 2024: 191). For these men, being active and perceiving themselves as healthy appeared to be an important counterpoint to dominant sociocultural perceptions of ageing as a period in which they are expected to ‘slow down’, and when their (physically inactive) bodies begin to decline limiting their ability to ‘age well’ (Pike 2015).

No less importantly, for the men who engaged in AB, ‘modes of care, friendship, and interdependence became central to their experiences’ (Thomas and Thurnell-Read 2024: 191) alongside their competitive involvement in walking football as part of ‘an essential process of negotiation as attempts to maintain masculine self-identity are put into dialogue with the realities of ageing’ (Thomas and Thurnell-Read 2024: 205). Indeed, although much of the initial appeal and motivation for engaging with AB was to become physically active (and often competitive) again, it was the inherently enjoyable and social nature of the programme (Bullough *et al*. 2023), and how it facilitated what the men perceived as male-appropriate banter and camaraderie with new like-minded peers (Sharp *et al*. 2024, Thomas and Thurnell-Read 2024), which was essential for their continued engagement with the programme. They developed new social networks and regularly met outside of formal programme sessions which became important parts of ongoing peer support, both when engaged in AB and outside the programme. In this regard, for these men, AB appeared pivotal in ‘creating and maintaining social connections by providing a place for men to connect with other men through shared experiences and interests’ (Sharp *et al*. 2024: 5). This was important not least because, in addition to the diverse experiences of men, many frequently encounter ‘challenges with establishing authentic and supportive peer relationships in their everyday lives’ (Sharp *et al*. 2024: 5) which has important implications for their physical and mental health.

The appeal of AB as a social context which cultivated traditional masculine norms and values (e.g. banter) and enabled men to improve their health (e.g. increased fitness, weight loss, and better diabetes management) by forming new social connections whilst ageing (Thomas 2024, Thomas and Thurnell-Read 2024). However, such norms and values can also be associated with health-harming and risk-taking behaviours (e.g. excessive alcohol consumption and problematic gambling) which shape how men perceive health and their willingness to seek help (Oliffe et al. 2020, Sharp *et al*. 2024, Galdas *et al*. 2025). Although none of the men in this study reported engaging in such behaviours, caution does need to be taken when adopting community-led approaches to health promotion, such as CCO-led programmes, which foster peer support for men and encourage traditional masculine norms and behaviours. As Sharp *et al*. (2024: 7) have noted in relation to mental health:

There is promising evidence that peer support can indeed align with masculine values and interests, albeit with careful consideration of the framing and delivery of these public health efforts. While peer support has been observed and applied in diverse contexts…there is considerable nuance in how it is received.

It is therefore critical that CCO-led health promotion programmes take into account not only the complexity and diversity of men’s experiences and preferences for traditional masculine values, but also how these can unintentionally negatively impact the health of men whilst also excluding them from potentially health promoting settings (Oliffe *et al*. 2020, Sharp *et al*. 2024, Thomas and Thurnell-Read 2024, Galdas *et al*. 2025).

In contrast, those men who only attended the LNI events and did not engage in AB were more likely to perceive age differently and tended to ‘align themselves with common scripts of deficit and deterioration’ (Thomas 2024: 5). For these men, being active was consistent with the ‘slowing down’ narrative associated with common perceptions of ageing which they appeared to have internalized and accepted (Pike 2011b, 2015, Thomas 2024). The internalization of these deficit narratives, and perhaps paradoxically for some, their experience of significant health events such as having a heart attack, led them to disengage in physical activity and meant they did not seek to engage in the programme. Perceptions of age-appropriateness were important in other ways, including the marketing of the programme and its characteristics, with competition, for example, being something which was considered to be more attractive to young people and not for those in in the upper age group (and beyond) for whom the programme was intended (Thomas 2024).

This having been said, while the sample of men who did not engage in AB was small, not all of them were physically inactive, had made ‘poor choices’, or failed to take sufficient personal responsibility to be active as a counter to declines in health which are commonly presented as inevitable features of ‘growing old’ (Pike 2015, 2019). Such neo-liberal and highly individualistic conceptions did not adequately capture the complexity of these men’s life experiences and circumstances which are often overlooked (Dionigi 2006, Dionigi and Son 2017, Thomas 2024, Thomas and Thurnell-Read 2024). Some of the men who did not engage in the programme were nevertheless active and reported exercising regularly in other settings following significant health scares (e.g. heart attack). Others, however, did not engage in AB and were not regularly physically active because of other overlapping and competing life demands which competed for their time and attention. These included including fulfilling family commitments and undertaking (often unpaid) caring responsibilities which they valued more highly than ‘finding time’ to be active by engaging in the AB programme. This diversity and complexity of experience should therefore caution us against accepting ‘individualized neoliberal conceptions of health and wellbeing…that overlook structural barriers to maintaining ‘good health’’ (Thomas and Thurnell-Read 2024: 205–206).

Our study has several strengths and limitations. One notable strength is that it provides what is to our knowledge the first set of empirical evidence which outlines how CCO-led health programmes can be a socially and culturally accepted setting in which to engage and improve the health of ageing men via a detailed analysis of the lived experiences of men who participate in such programmes. Any analysis of the impact of CCO-led programmes which are intended to promote the health of men should therefore necessarily explore the complex interrelationships between their practices and experiences of being active (or not), their health, and how these intersect with the masculine identities and practices of ageing men (Thomas 2024, Thomas and Thurnell-Read 2024). However, our sample of men were all self-defined as White, heterosexual, nondisabled, and only six men who did not engage in the AB programme consented to engage in semistructured interviews. More research is therefore needed to understand the wider generalizability of the experiences recalled here for other more diverse subgroups of men who engage in CCO-led programmes. This would appear a fruitful line of enquiry for, as we have been able to show with a sample of 17 men, there is some important commonality but also diversity in how they encounter physical activity and health in relation to ageing.

Exploring the intersectional experiences and interpretations more diverse groups of men (including those experiencing poverty, disabled men, and men from minority ethnic and lesbian, gay, bisexual, transgender, queer, intersex, asexual (LGBTQIA+) communities) have of masculinity, ageing and health in other types of community programmes and settings is crucial if we are to better understand how these contribute to, and are shaped by, gendered health inequalities (Galdas *et al*. 2025 ). Such work also helps to develop more culturally nuanced understandings of masculine norms which variously shape the effectiveness of engaging men in gender-responsive health promotion, harm-reduction approaches, and programmes underpinned by strengths-based healthy masculinities (Galdas *et al*. 2025). Indeed, as Galdas *et al*. (2025: e848) have argued, approaches that ‘focus narrowly on male-friendly programmes—designed to appeal to stereotypical masculine interests or behaviours—might achieve short-term success but rarely sustain these improvements amid the risk of reinforcing rigid gender norms’.

## CONCLUSION

This study examined how age, and perceptions of ageing, helped shape whether physically inactive men living in under-served communities engaged, or did not engage, in the CCO-led health programme, AB. The findings demonstrate the need to understand the diversity and complexity of men’s experiences for, while the AB programme represented ‘an outlet for embracing ageing and resisting popular deficit configurations of ageing’ (Thomas 2024: 4) by engaging in physical activity such as walking football, others were more likely to internalize and accept more dominant sociocultural perceptions of ageing. The findings also pointed towards the enduring impact of the wider structural and cultural barriers which continue to impact some men’s ability to be physically active, and in this regard, it is essential that future studies do not overlook the continued significance of the social determinants of health for men’s engagement in CCO-led programmes and their impact on health and wellbeing (Dionigi 2006, Dionigi and Son 2017, Thomas 2024, Thomas and Thurnell-Read 2024). These determinants are clearly beyond the scope and direct reach of individual CCO-led health promotion programmes which might make an important contribution to the promotion of physical activity and health among men, but which cannot reasonably be expected to overcome the deep-seated inequalities and inequities which underpin those very same determinants (Galdas *et al*. 2023, 2025).

Our findings also have other important practical and policy implications which are informed by existing frameworks (see Galdas *et al*. 2023, 2025). For example, CCOs should consider developing community-based, relational, and gender-transformative approaches to programme design and delivery which account for diverse and context-dependent masculine norms (Galdas *et al*. 2023, 2025). Such approaches are most effective when they are underpinned by conceptions of positive, strengths-based, healthy masculinities which reinforce notions of peer support, camaraderie and openness (Galdas *et al*. 2025), and when they capitalize on the ability of CCOs to engage men through their association with the brand of professional sports organizations (Pringle *et al*. 2013, 2021, Wilcock *et al*. 2021, Brazier *et al*. 2024, Maclean *et al*. 2025). Developing co-designed programmes, which make use of existing nonclinical community-based assets, and which involve the delivery of nonthreatening, solutions-focused, activities by relatable practitioners in the communities where men live and work (Robertson *et al*. 2016, Wilcock *et al*. 2021, Galdas *et al*. 2025) are other important programme design principles which might be considered by CCOs. Equally important is the need to develop adequately resourced, contextually-relevant, gender-sensitive approaches to programme monitoring and evaluation. These approaches should be concerned with identifying the intended and unintended outcomes of programmes, and what works, for whom, in what settings, how, and with what outcomes (Brazier *et al*. 2024, 2025a). Given the challenges CCOs can often face in these areas, the development of authentic, meaningful and mutually beneficial collaborations with evaluation partners can support effective programme design, and their systematic monitoring and evaluation, especially when this is underpinned by ‘research in men’s lived experiences as well as the epidemiological profiles embedded in population and public health data (inclusive of men’s health services usage)’ (Galdas *et al*. 2025: e852).

## Data Availability

The participants did not consent to their data being shared more widely (other than for the agreed purposes of publication), so no other data are available.
